# Parkinson’s Disease Blood Test for Primary Care

**Published:** 2022-07-22

**Authors:** Sid E. O’Bryant, Melissa Petersen, Fan Zhang, Leigh Johnson, Dwight German, James Hall

**Affiliations:** 1 Department of Neuroscience and Pharmacology, University of North Texas Health Science Center, Fort Worth, Texas, USA; 2 Department of Medicine, University of North Texas Health Science Center, Fort Worth, Texas, USA; 3 Department of Psychiatry, UT Southwestern Medical School, Dallas, Texas, USA

**Keywords:** Serum, Parkinson’s disease, Primary care, Screening tool, Blood test

## Abstract

**Background:**

A blood-test that could serve as a potential first step in a multi-tiered neurodiagnostic process for ruling out Parkinson’s disease (PD) in primary care settings would be of tremendous value. This study therefore sought to conduct a large-scale cross-validation of our Parkinson’s disease Blood Test (PDBT) for use in primary care settings.

**Methods:**

Serum samples were analyzed from 846 PD and 2291 volunteer controls. Proteomic assays were run on a multiplex biomarker assay platform using Electrochemiluminescence (ECL). Diagnostic accuracy statistics were generated using area under the receiver operating characteristic curve (AUC), Sensitivity (SN), Specificity (SP) and Negative Predictive Value (NPV).

**Results:**

In the training set, the PDBT reached an AUC of 0.98 when distinguishing PD cases from controls with a SN of 0.84 and SP of 0.98. When applied to the test set, the PDBT yielded an AUC of 0.96, SN of 0.79 and SP of 0.97. The PDBT obtained a negative predictive value of 99% for a 2% base rate.

**Conclusion:**

The PDBT was highly successful in discriminating PD patients from control cases and has great potential for providing primary care providers with a rapid, scalable and cost-effective tool for screening out PD.

## Introduction

Parkinson’s disease (PD) is the second most common neurodegenerative disease affecting over 1% of individuals over the age of 65 in the United States [1]. The cost of PD to our society was reported to be $23 billion annually in the U.S. in 2005 [2]. Given the rapidly growing segment of the elderly population, these costs will continue to increase over the next several decades. The most accurate diagnosis of PD comes from specialty clinics where clinical assessments and advanced neurodiagnostic procedures are costly, time-consuming, and invasive. In the United States, primary care clinics serve as the “gatekeeper” to specialty clinics and these frontline primary care practitioners provide the referrals for advanced diagnostic procedures. However, the average duration of primary care visits is around 18 minutes making detailed neurological examinations difficult [3].

In 2017, Plouvier and colleagues interviewed community-dwelling PD patients and General Practitioners (GPs) to understand their thoughts on the role of primary care in PD management. These authors found discrepancies between patients’ and GP views as patients felt that GPs lacked expert knowledge or skills and diminished the role of GPs in patients at advanced PD stages. GPs, on the other hand, valued patient autonomy in early-stage decision making but in more advanced PD stages felt a more active role of the GP is warranted. The authors stated that patients would likely benefit from the more holistic approach brought by the GP if done in conjunction with specialty care [4].

Currently, however, there are no rapid or cost-effective tools for primary care providers to use in daily practice to screen patients with possible PD symptoms. Within primary care settings, the purpose of screening tests is to rule out patients who do not require additional medical procedures or diagnostic follow-up, thereby resulting in stress reduction and cost containment.

Our team has proposed a multi-tiered neurodiagnostic process for neurodegenerative diseases including Alzheimer’s disease, Parkinson’s disease and Dementia with Lewy Bodies (DLB) [5–7].

Over the last several decades, the search for biomarkers that have diagnostic and prognostic utility in neurodegenerative diseases has grown exponentially with the majority of work focusing on neuroimaging and cerebrospinal (CSF) methodologies. In fact, the dopamine transporter single photon emission CT [DaT-SPECT] has been approved as a tool for diagnosing PD. Research suggests that CSF markers may also hold utility in the differential diagnosis of neurodegenerative diseases [8–15]. While advanced neuroimaging and CSF methods have tremendous potential as biomarkers of PD, invasiveness, accessibility and cost barriers preclude these from being utilized as an initial step in detection procedures. Therefore, it has been proposed that blood-based biomarker methods may serve as the optimal first step in a multi-tier detection process [17,18] and requires additional investigation, similar to their application in the field of oncology [16–21]. Our team has conducted a series of studies demonstrating the utility of blood-based biomarkers for detecting PD as well as discriminating PD from other neurodegenerative diseases [22]. Here we completed a large-scale cross-validation of our PD Blood Test (PDBT) for use in primary care settings.

## Materials and Methods

### Participants and reference database

Parkinson’s disease data: Our team recently completed baseline and longitudinal assays on serum samples from the previously conducted DATATOP trial. DATATOP methods regarding participant recruitment, study design, enrollment, consent procedures, and funding sources have all been previously published. Briefly, DATATOP was a multi-site placebo- controlled clinical trial designed to test the impact of deprenyl 10 mg/d and/or tocopherol (vitamin E) 2000 IU/d on PD progression (in combination with levodopa) [23]. A total of 656 baseline PD serum samples had requisite data in our database, and were used in the current study. An additional n=190 serum samples from PD cases were already included in our research database from PD specialty evaluations. Therefore, there was a total number of n=846 PD cases. No cases included in this study had a diagnosis of PD-dementia.

Neurodegenerative Disease Blood Test Reference Database (NDRD): Complex diseases, such as neurodegenerative diseases, require that multiple factors (or biological pathways) be considered when making a diagnosis rather than just a single factor. In our prior work, we have generated a blood test for detecting AD specifically for use in primary care settings [5,22,24–26]. This blood test was discovered and validated on the premise that taking multiple biomarkers into account would yield a more accurate approach than any single marker [22,25,26]. This multi-marker approach has led to multiple in vitro diagnostic (IVD) tests being advanced to clinical use in the field of oncology. However, in order advance such an “algorithm” to the clinic it requires an appropriate Reference Database that, in practice, when combined with the algorithm itself would be covered under FDA regulations as Software as a Medical Device (SAMD) [24–27].

Therefore, our team generated and published a NDRD. The NDRD contains data from n>5000 participants across a broad range of diseases (e.g., AD, PD, DLB, controls) and blood fractions (serum and plasma). Only completely de-identified data are included in the NDRD. To be included in the NDRD, the data came from studies that

1. Conducted comprehensive cognitive assessments on all participants for accurate diagnosis and
2. Conducted under IRB approval and written informed consent was obtained.

#### Controls:

Controls in the database had no neurodegenerative disease diagnosis, performed within normal cognitive parameters on neuropsychological testing and reported no decline in activities of daily living. For the purpose this study, control samples from serum data were utilized (controls n=2,291).

### Proteomics

All serum samples were assayed in the University of North Texas Health Science Center Institute for Translational Research (ITR) Biomarker Core. The ITR Biomarker Core utilizes the Hamilton Robotics Easy Blood for blood processing, aliquoting, and realiquoting. A custom Hamilton Robotics StarPlus system was utilized for the preparation of all plates. Proteomic assays were run on a multiplex biomarker assay platform using electrochemiluminescence (ECL) per our previously published methods using commercially available kits [28]. ECL technology uses labels that emit light when electronically stimulated, which improves the sensitivity of detection for many analytes even at very low concentrations. ECL measures have well established properties for being more sensitive and requiring less volume than conventional ELISAs, the gold standard for most assays. We recently reported the analytic performance of several proteins for n>1,300 samples across multiple cohorts and diagnoses (normal cognition, mild cognitive impairment, and AD) [22]. The assays are reliable and in our experience with these assays show excellent spiked recovery, dilution linearity, coefficients of variation, as well as detection limits. Inter and intra-assay variability has been excellent. Internal QC protocols are implemented in addition to manufacturing protocols including assaying consistent controls across batches and assay of pooled standards across lots. A total of 500 μl of serum was utilized to assay (singlicate) the following markers: Fatty Acid Binding Protein (FABP)-3, Beta 2 Microglobulin (B2M), Pancreatic Polypeptide (PPY), C-Reactive Protein (CRP), ICAM-1, thrombopoietin, α2 Macroglobulin (A2M), exotaxin 3, tumor necrosis factor alpha (TNF-α), tenascin C, Interleukin (IL)-5, IL-6, IL-7, IL-10, IL-18, I-309, Factor 7 (Factor VII), Vascular Cell Adhesion Molecule 1 (VCAM 1), TARC and Serum Amyloid A (SAA). Our lab has run n>20,000 of these assays over the last several years with all CVs being <10% with the majority being <=6%.

### Statistical analysis

Statistical analyses were conducted using the R (V3.3.3) statistical software, SPSS 24 (IBM), and SAS. Support Vector Machine (SVM) analyses were conducted to discriminate PD cases from controls. SVM is based on the concept of decision planes that define decision boundaries and is primarily a classifier method that performs classification tasks by constructing hyperplanes in a multidimensional space that separates cases of different class labels.

Diagnostic accuracy was calculated via Receiver Operating Characteristic (ROC) curves. The sample was randomly split (70/30) into training and test samples with diagnostic accuracy derived from the test sample. Finally, to provide estimates of the overall utility of the PDBT in ruling out PD in primary care settings, negative predictive values (NPVs; the probability that subjects with a negative screening test truly do not have disease) were calculated using a range of base rates including 2%, 5%, 10% and 15%.

## Results

Descriptive statistics of the sample are provided in Table 1. The average age of the sample 63.8 (SD=13.4). The PD group was younger, more likely to be male, and reported higher levels of education (p-values<0.001) as compared to the normal control group.

**Figure 1:**
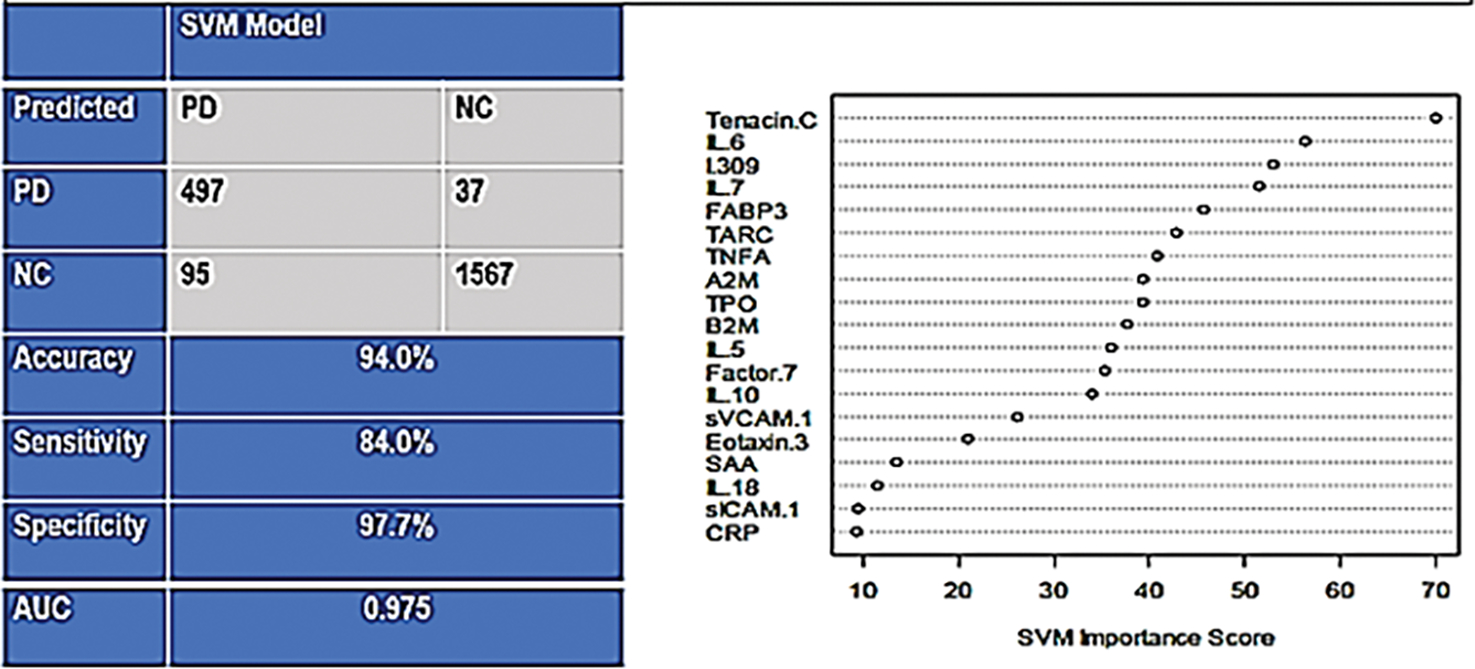
Support Vector Machine (SVM) based output for the Parkinson’s disease Blood Test with reported findings for the accuracy and variable importance plot for the training set.

In the training sample, there were a total 592 PD samples and 1604 control samples. The SVM was applied with a 5-fold internal cross-validation within the training sample for initial analysis and internal validation. The PDBT yielded an AUC of 0.98 with a SN of 0.84 and SP of 0.98 within the training set. The overall classification accuracy (correct and incorrect) along with the diagnostic accuracy statistics and variable importance plot in Figure 1.

**Figure 2:**
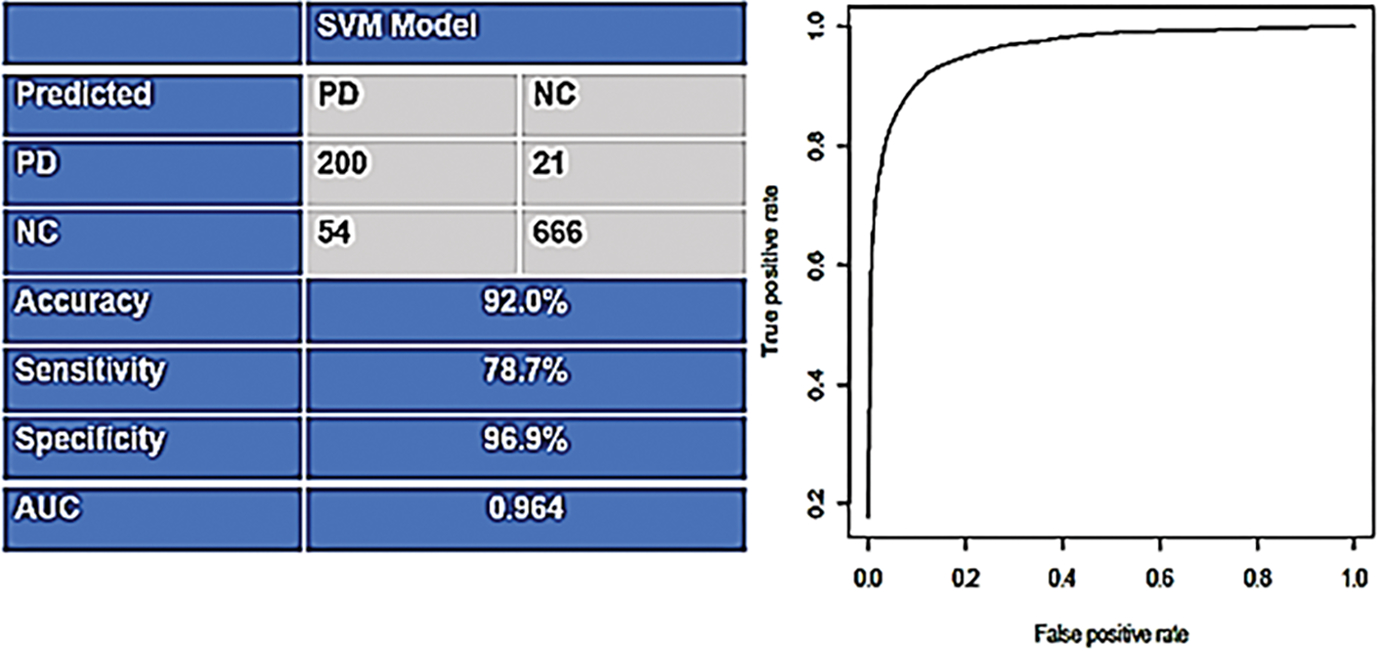
Support Vector Machine (SVM) based output for the Parkinson’s disease Blood Test with reported findings for the accuracy and receiver operating characteristics curve for the test set.

Next the PDBT was directly applied to the test sample, which consisted of n=254 PD cases and n=687 controls. The PDBT yielded an AUC of 0.964 with a SN of 0.79 and SP of 0.97. The classification accuracy (correct and incorrect) as well as the ROC curve shows in Figure 2.

Finally, to provide a sense of how the PDBT would perform as a screening tool for ruling out PD in primary care settings, the NPV was calculated for a range of base rates. With a 2% base rate, the NPV was 0.99. Therefore, the physician is 99% accurate in ruling out PD with a negative blood test. The NPV for 5%, 10% and 15% base rates were 99%, 98% and 96%, respectively. If a physician used a 5% base rate for those adults complaining of new onset motor changes, and saw 5000 patients, the PDBT would rule out 4,660 patients from needing any additional testing procedures. There would be only 53 false negative cases.

## Discussion

The current data provide additional support for the utility of the PDBT in primary care settings. In the current study, data were pooled for an aggregate sample of 846 PD samples and 2,291 control samples. Overall, the accuracy of the PDBT is excellent (i.e., >98%) for ruling out disease. As we have previously published, the goal of a screening test in primary care settings for neurodegenerative diseases is to rule out the disease [10,29], which is consistent with the use and performance of the vast majority of screening tests used in primary care settings on a daily basis.

In addition to detecting PD, our team has conducted a series of studies examining the possibility of a PDBT in discriminating PD from other neurodegenerative diseases. In an initial study, we analyzed our proteomic profile from 349 patients (150 AD, 49 PD, and 150 controls) and found that our serum-based PDBT-proteomic profile was >98% accurate in discriminating PD from AD. In the next study, we examined a plasma proteomic profile from 145 patients (32 PD, 57 DLB, 56 controls) from the Mayo Clinic, Jacksonville Alzheimer’s Disease Research Center and Movement Disorders Clinic. The blood-based proteomic profile was highly accurate in detecting neurodegenerative disease yielding an AUC of 0.94 versus controls, in discriminating PD from DLB with an AUC of 0.84, as well as discriminating AD/DLB from non-demented PD (AUC of 0.98) [7]. Next, we conducted a study in the Harvard Biomarker Study Bio repository by assaying n=150 plasma samples (PD n=50, “other neurodegenerative disease” n=50 [AD n=12, FTD n=25, other n=13], control n=50). The proteomic profile approach was highly accurate in discriminating PD from other neurodegenerative diseases with an AUC of 0.98. Therefore, this prior work suggests that our approach cannot only detect PD, but that it can also discriminate PD from other neurodegenerative diseases. The latter would be of value to primary care practitioners to know the appropriate referral for a given patient as well as for general neurology clinics when receiving referrals to determine the most appropriate clinician to receive the new patient.

There are limitations to the current study. First, it is possible that additional proteomic markers, not examined in this study, will increase the overall accuracy of the PDBT. AD specific markers such as Amyloid Beta (Aβ) 40, Aβ 40, tau and neurofilament light chain (NfL) have been increasingly explored both in blood and CSF for their utility in detecting AD and PD as well as distinguishing between neurodegenerative conditions [29–35]. Karikari and colleagues examined phosphorylated tau 181 (ptau181) and found that this one marker alone reached an AUC of 0.81 in distinguishing AD from PD [36]. In addition to AD specific biomarkers, PD specific biomarkers such as a-synuclein have also shown promise particularly when applied to distinguishing PD from other related conditions such as DLB [30,37]. While increased accuracy is not needed for the current screening Context of Use (COU), increased accuracy would be needed for the generation of a blood-based diagnostic test and therefore the addition of other such markers should be considered in future work. Second, it is possible that novel or known genetic markers will improve the diagnostic accuracy. It is of importance to note that the COU for the PDBT is not diagnostic, but rather as a screening tool to rule out PD within primary care settings. It will be important for this work to be replicated to ensure reproducibility.

## Conclusion

The availability of the PDBT for primary care holds tremendous benefit. First, this is a rapidly scalable technology that can be implemented globally as a Laboratory Developed Test (LDT). The PDBT would provide primary care providers with actionable and objective information that is supported by several studies and many patients. Additionally, it is thought that the earlier therapeutics can be administered, the more beneficial they are to patients. The availability of the PDBT in primary care settings would provide a tool for rapid referrals. Finally, for clinical trials, the PDBT would provide a means of drastically expanding access to screening procedures well beyond specialty clinics. Overall, our series of studies, in combination with the current results, strongly support the utility of the PDBT for the COU of screening out PD in primary care settings.

## Figures and Tables

**Table 1:** Demographic characteristics of the cohort.

	PD	Normal control	p-value
	Mean(SD)	Mean(SD)	
N	846	2291	
Age	59.5(12.6)	65.4(13.4)	4.16E-29
Education	13.76(4.65)	12.74(6.62)	1.59E-06
Gender (%M)	62.8	40.1	5.65E-30

## Abbreviations

A2M: α2 Macroglobulin
AD: Alzheimer’s Disease
AUC: Area Under the Curve
B2M: Beta 2 Microglobulin
COU: Context of Use
CRP: C-Reactive Protein
DATATOP: Deprenyl And Tocopherol Antioxidative Therapy of Parkinsonism
DaT-SPECT: Dopamine Transporter Single Photon Emission CT
ECL: Electrochemiluminescence
ELISA: Enzyme-Linked Immunoassay
Factor VII: Factor 7
FABP: Fatty Acid Binding Protein
GPs: General Practitioners
IVD: In vitro Diagnostic
ITR: Institute for Translational Research
ICAM-1: Intercellular Adhesion Molecule 1
IL: Interleukin
LBD: Lewy Body Dementia
NPV: Negative Predictive Value
NDRD: Neurodegenerative Disease Blood Test Reference Database
PPY: Pancreatic Polypeptide
PD: Parkinson’s Disease
PDBT: Parkinson’s Disease Blood Test
ROC: Receiver Operating Characteristic
SN: Sensitivity
SAA: Serum Amyloid A
SAMD: Software as a Medical Device
SP: Specificity
SVM: Support Vector Machine
TARC: Thymus and Activation-Regulated Chemokine
TNF-α: Tumor Necrosis Factor-Alpha
VCAM 1: Vascular Cell Adhesion Molecule 1
